# *n*-Butyl Cyanoacrylate Synthesis. A New Quality Step Using Microwaves

**DOI:** 10.3390/molecules19056220

**Published:** 2014-05-15

**Authors:** Yaquelin Ramos Carriles, Rubén Álvarez Brito, Ricardo Martínez Sánchez, Elayma Sánchez Acevedo, Paola Rodríguez Domínguez, Wolf-Dieter Mueller

**Affiliations:** 1 Chemistry-Physics Department, Faculty of Chemistry, University of Havana, Zapata and G, Vedado, Havana 10400, Cuba; E-Mails: ruben@fq.uh.cu (R.Á.B.); elayma_sanchez@fq.uh.cu (E.S.A.); paola_rodriguez@fq.uh.cu (P.R.D.); 2 Polymers Laboratory, IMRE, University of Havana, Zapata and G, Vedado, Havana 10400, Cuba; E-Mail: ricardo@imre.oc.uh.cu; 3 Charité-Universitaetsmedizin Berlin, Assmannshauser Str.4-6, Berlin 14197, Germany; E-Mail: Wolf-Dieter.Mueller@charite.de

**Keywords:** *n*-butyl cyanoacrylate, poly (*n*-butyl cyanoacrylate), microwaves irradiation

## Abstract

Alkyl cyanoacrylates are interesting products for use in industry because of their properties enabling them to stick together a wide range of substrates. *n*-Butyl cyanoacrylate is one of the most successfully used tissue adhesives in the field of medicine because it exhibits bacteriostatic and haemostatic characteristics, in addition to its adhesive properties. At present, its synthesis is performed with good yields via Knoevenagel condensation using conventional sources of heating, but this requires a long processing time. The aim of this work was to look for a new way of synthesising *n*-butyl cyanoacrylate using microwave irradiation as the source of heating. This non-conventional source of heating most likely reduces the process time of the synthesis. In comparison with a conventional heating source, such as an oil bath, the results showed the advantages of this method whereby the *n*-butyl cyanoacrylate gave the same yield and quality with a reduction in the reaction time by a factor of 3-5-fold.

## 1. Introduction

Alkyl cyanoacrylates (CA) were discovered in the 1940s as a result of research on transparent polymer materials for military use [1]. These products have the property of enabling a wide range of substrates to stick together and are therefore very interesting for industrial applications. Some of them have, moreover, a huge impact in the medicine field. *n*-Butyl cyanoacrylate (BCA) is one of the most successfully used tissue adhesives [2,3,4,5,6], because it provides great tension resistance with bacteriostatic and haemostatic characteristics [7,8]. It is also used as precursor for nanoparticle preparations for controlled delivery of drugs [9,10,11].

The most commonly applied synthesis of CA is the Knoevenagel condensation between the corresponding alkyl cyanoacetate and formaldehyde in the presence of a basic catalyst and a solvent capable of removing the condensation water by azeotropic distillation. The monomer is not formed in one step, but is instead obtained from the pyrolysis of the corresponding alkyl polycyanoacrylate (PCA). Two procedures are possible to achieve this aim, namely the direct pyrolysis of the oligomer mixture (the most common approach), or the isolation and purification of the mixture previous to its pyrolysis [12,13,14]. In this work, these procedures are termed “direct synthesis” and “indirect synthesis”, respectively. Conventional sources of heating have been used for these syntheses. Although the yields are high, the reaction times required to attain them are quite long.

Microwave irradiation is a non-conventional heating source used in many organic syntheses because it frequently provides a considerable reduction in the reaction time. One example of organic synthesis assisted by microwaves, is the Knoevenagel condensation, and the results are very good [15,16,17]. Moreover, some of these reactions are eco-friendly too, because they are carried out using inorganic supports that can be reused in other reaction cycles and avoid the use of solvents [18,19,20].

The aim of this work was to synthesize BCA using microwave irradiation, and to compare the results with those obtained from a classical synthesis using an oil bath.

## 2. Results and Discussion

The BCA synthesis occurs in three steps. In the first, the monomer is obtained, but it polymerizes and oligomers are formed due to the conditions of the reaction medium (second step).

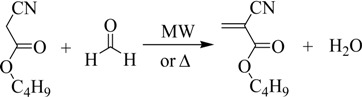
(1)

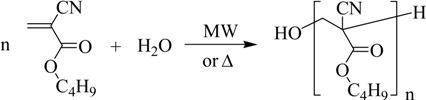
(2)


Consequently, the third step is intended to depolymerize the oligomers previously obtained:

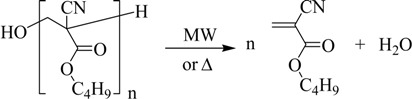
(3)


**Figure 1:**
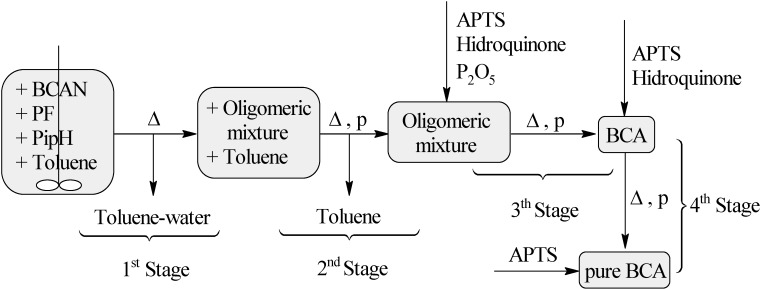
*n*-Butyl cyanoacrylate synthetic stage sequence when using oil bath heating (∆). BCAN = *n*-butyl cyanoacetate, PF = paraformaldehyde, PipH = piperidine hydrochloride.

One can see in Table 1 the results obtained in the “direct synthesis” using an oil bath, whose stages are represented in Figure 1. Table 1 also includes the values of the results through the use of a microwave oven, which diminishes the duration of the “direct synthesis”.

**Table 1:** Results of BCA *direct synthesis* by an oil bath and by a microwave oven. Exp. = Experiments.

Heating Source	Exp.	Time (min)				Total Time (min)	Monomer Yield (%)
		1st Stage	2nd Stage	3th Stage	4th Stage		
Oil bath	1	40	36	60	23	159	72
	2	48	17	57	17	139	63
	3	40	43	87	20	190	67
*Mean values*						*163 ± 26*	*67 ± 5*
Microwave oven	1	24	-	15	-	39	72
	2	9	-	38	-	47	63
	3	10	-	27	-	37	61
*Mean values*						*41 ± 5*	*65 ± 6*

**Figure 2:**
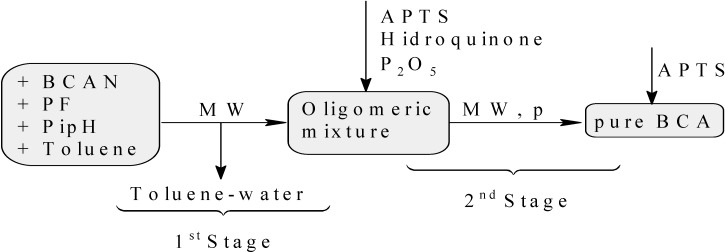
Direct synthesis of BCA assisted by microwave irradiation (MW).

It is necessary to emphasize that comparing the second process (using a microwave oven) with the first one, some stages are avoided. This means that the first and the second stage come together (without stirring and reduced pressure), and the fourth stage is totally eliminated (Figure 2). The reason for this is because it is possible to obtain the final product with the same quality as in the case when using an oil bath when reaching the third stage. Therefore, when a microwave oven is used, only two stages are needed: toluene-water extraction and oligomers pyrolysis.

Table 1 indicates that the reaction time was reduced by a factor of 3–5 in the “direct synthesis” assisted by a microwave, in comparison with an oil bath heating, while the yield did not change significantly.

The oligomers obtained by Knoevenagel condensation were separated and purified in the “indirect synthesis” to depolymerize them by reducing the pressure using an oil bath or a microwave oven. The results are shown in Table 2. The reaction time was reduced, as in the case of “direct synthesis”, but there has not been a favorable change in the yield when microwave irradiation was used.

**Table 2:** Results of BCA *indirect synthesis* (depolymerization stage).

Heating Source	Exp.	Time (min)	Yield (%)
Oil bath	1	75	81
	2	58	76
	3	65	65
*Mean values*		*66 ± 9*	*74 ± 8*
Microwave oven	1	40	85
	2	37	72
	3	51	50
*Mean values*		*43 ± 7*	*69 ± 18*

The decrease in the reaction times caused by the use of microwave irradiation is a known fact in organic synthesis, and our results are not an exception. Concerning the yields, it is necessary to make a distinction between the corresponding steps of BCA syntheses [Equations (1) and (2)]. Water and BCA are produced in the first step, but the BCA polymerizes *in situ*. The second stage, the pyrolysis, is the reason why the mean global yield was 67% (oil bath) and 65% (microwave oven) in the “direct synthesis”. It was possible to verify that the thermic degradation yield of the pure oligomers in “indirect synthesis” using an oil bath and a microwave oven was similar to that obtained by “direct synthesis” (Table 2). This means that, the monomer yield is independent of the heating method. It depends on the degradation product characteristics. According to Chorbajiev *et al.* [21]: “the thermal degradation of low molecular poly(cyanoacrylates) is a chain process, starting at the ends of polymer chains. Volatile monomeric alkyl-α-cyanoacrylates are formed and the product of degradation is the corresponding dialkyl-α,α´-dicyanoglutarate, which decomposes at higher temperatures”. The use of microwave irradiation does not change this behavior and it indicates that the yield cannot be higher than that reported in Table 1 and Table 2.

## 3. Experimental Section

### 3.1. General

Paraformaldehyde, phosphorus pentoxide and acetone (Panreac, Barcelona, Spain), toluene (QUINSA, D.F., Mexico), piperidine hydrochloride (Acros Organics, Geel, Belgium), monohydrated *p*-toluene sulfonic acid (APTS), and tetrahydrofuran (THF) (Merck Schuchardt OHG, Hohenbrunn, Germany), hydroquinone Analar (BDH Chemicals Ltd, Poole, England), and ethanol (96%) (national production) were used without further purification. *n*-Butyl cyanoacetate (Probus, Barcelona, Spain) was distilled previous to its use. The Fourier transform IR spectra were measured using a JASCO FT/IR-4100 spectrometer and KBr tablets. ^1^H-NMR spectra were obtained at 250 MHz using a Bruker 250 Avance NMR spectrometer with tetramethylsilane as reference. Microwave radiation was carried out using a microwave Milestone’s START System. The BCA synthesis occurs fundamentally in two steps (Equations (1) and (2)). To check the reproducibility of the methods, three experiments were carried out applying both heating sources in the cases of “direct” and “indirect synthesis”.

### 3.2. Direct Synthesis of BCA Using an Oil Bath

*n*-Butyl cyanoacetate (50 mL, 0.3517 mol), paraformaldehyde (11.689 g, 0.3 mol), piperidine hydrochloride (0.3 g, 0.002 mol) and toluene (37 mL, 0.3 mol) were mixed in a three-neck round-bottom flask. The mixture was heated to about 130 °C under stirring, while distilling out the water and toluene. When the distillation stopped, the mechanical stirring was removed and the rest of the water-toluene mixture extracted under reduced pressure. The reaction ended after about 75 min, when distillation stopped. The formed water was measured. Phosphorus pentoxide (1.4218 g, 0.01 mol), hydroquinone (0.4405 g, 0.004 mol) and APTS (0.4517 g, 0.002 mol) were then added to the product, which was pyrolyzed for one hour at 109 °C between 0.15–1 mm Hg. Hydroquinone (0.1767 g, 0.002 mol) and APTS (0.0961 g, 0.0005 mol) were then added to the obtained monomer, which was purified by distillation. APTS (0.02 g, 0.0001 mol) was added to the final product (36 mL, 67%). The distillation equipment used in the last two stages had been previously treated with a 20% APTS solution. Figure 1 shows all these stages.

*BCA*: IR (KBr): 3124, 2960–2878, 2235, 1735, 1612, 1461, 1283-1187, 1386 cm^−1^. ^1^H-NMR (acetone-d_6_, 250 MHz): *δ* 0.96 (3H, m_3_, *J* = 7.23 Hz, (CH_2_)_3_-*CH_3_*), 1.44 (2H, m_6_, *J* = 8.03 Hz, (CH_2_)_2_-*CH_2_*-CH_3_), 1.72 (2H, m_5_, *J* = 7.76 Hz, CH_2_-*CH_2_*-CH_2_-CH_3_), 4.29 (2H, m_3_, *J* = 7.23 Hz, O-*CH_2_*-(CH_2_)_2_-CH_3_), 6.63 (1H, s,=CH ), 7.06 (1H, s, =CH).

### 3.3. Direct Synthesis of BCA Assisted by Microwave

The same quantities of reactants used in the previous synthesis were mixed in a round bottom flask coupled with a Dean-Stark apparatus. The mixture was irradiated in a multimode microwave oven with 200, 250 and 650 W providing temperatures of 90, 100 and 200 °C, for 5, 15 and 4 min respectively, when distillation of the water-toluene azeotrope ended. The formed water was then measured. The Dean-Stark apparatus was replaced by reduced pressure distillation equipment, which had been previously treated with an APTS solution. The pyrolysis was performed for 15 min at 200 °C with 700 W at 17 mm Hg (see Figure 2). The monomer thus obtained (39 mL, 72%) did not require any purification, since it exhibited the same appearance and sticking power as the pure monomer obtained using an oil bath. Its ^1^H-NMR and FT-IR spectra displayed the same signals reported above.

### 3.4. Indirect Synthesis of BCA Using an Oil Bath and a Microwave Oven

In this procedure, we used the oligomeric mixture obtained in the BCA “direct synthesis” (at the end of the second stage when using an oil bath and at the end of the first step in the case of using a MW). The oligomeric mixture was dissolved in THF, precipitated in water and dried. The purified *n*-butyl polycyanoacrylate (PBCA) was analyzed by FT-IR and ^1^H-NMR spectroscopy. Then, PBCA (50.2818 g, 0.3283 mol relative to the monomer unit) was mixed with phosphorus pentoxide (1.3682 g, 10^−2^ mol), hydroquinone (0.4108 g, 0.004 mol) and APTS (0.4045 g, 0.002 mol). The mixture was pyrolyzed for 66 min when an oil bath was used and 34 min in the case of microwave irradiation. The yields were 81% for an oil bath and 72% for microwave.

*PBCA*: IR (KBr): 2962–2874, 2248, 1748, 1464, 1384, 1253 cm^−1^. ^1^H-NMR (acetone-d6, 250 MHz): δ 0.98 (3H, m, (CH_2_)_3_-*CH_3_*), 1.52 (2H, m, (CH_2_)_2_-*CH_2_*-CH_3_), 1.76 (2H, m, CH_2_-*CH_2_*-CH_2_-CH_3_), 2.73 (2H, m, CH_2_ backbone), 4.28 (2H, m, O-*CH_2_*-(CH_2_)_2_-CH_3_).

## 4. Conclusions

BCA was synthetized using microwave radiation and an oil bath as heating sources. The best synthetic method was “direct synthesis” assisted by microwaves, because yields were acceptable (65.3% ± 5.9%), reproducibility was good, and the reaction times were greatly reduced in comparison with the conventional oil bath heating. In addition to this improvement, it is important to emphasize that the use of microwaves is more straightforward and much safer than an oil bath.
